# Urological Care and COVID-19: Looking Forward

**DOI:** 10.3389/fonc.2020.01313

**Published:** 2020-07-21

**Authors:** Tommaso Prayer-Galetti, Giovanni Motterle, Alessandro Morlacco, Francesco Celso, Deris Boemo, Massimo Iafrate, Filiberto Zattoni

**Affiliations:** ^1^Clinica Urologica, Department of Surgical and Oncological Sciences, University of Padua, Padua, Italy; ^2^Management Health Services Department, University Hospital of Padua, Padua, Italy

**Keywords:** COVID-19, management, organization, surgery, urology

## Abstract

The recent COVID-19 pandemic represents a worldwide emergency and it is affecting healthcare at every level, including also urological care and especially oncologic patients. Recent epidemiological models show that, without effective treatment or vaccine, there will be a long-lasting phase of cohabitation with the virus. Current experts' opinions recommend performing only non-deferrable uro-oncological surgery and postponing other activities until the end of the emergency, with particular concerns regarding the safety laparoscopy. Veneto Region and Padua Province represent one of the first site of the pandemic spread of the virus outside China, thus we present our experience as a Urological Referral Center in applying a segregated-team work model of organization during the month of March 2020, with a stratified organization of activities, adequate screening and protection for patients and staff were adopted. Compared to the same period of last year even if a 19.5% reduction was experienced in overall surgical activity while maintaining a comparable proportion of oncologic robotic and laparoscopic surgery and guaranteeing care also for high priority non-oncological patients. No cases of COVID-19 infection were reported in staff members nor in patients and the number of surgical complications was comparable to that of last year. Therefore, in our opinion the recommended significant reduction in urological care, including surgical activities, is likely unrealistic in the long period with unknown effects affecting mostly oncological patients. Our experience introducing a segregated-team work model might represent a model for future planning.

## Introduction

### Background

The recent outbreak of severe atypical pneumonia, which started in December 2019 in the city of Wuhan in the Hubei province of China, has been associated with a previously unknown coronavirus, SARS-CoV-2. In few weeks, the rapid and uncontrolled spread of SARS-CoV-2 became a pandemic (1), involving more than 100 countries worldwide. The highly contagious SARS-CoV-2 causes a severe acute respiratory syndrome, named COVID-19, with not negligible morbidity and mortality.

To date, besides containment measures and social distancing, we do not have any effective treatment or vaccine against COVID-19; as a consequence, several governments issued regulations restricting non-urgent medical and surgical activities, including some kind of oncological patients.

Unfortunately, it is likely that SARS-CoV-2 and other similar viruses will keep circulating in the human host for near future; recent projections reported that, without other interventions, social distancing may be necessary until 2022 with late contagion possible as late as 2024 (2).

In light of these considerations, urologists must be aware that not only daily practice changed in this period, but also will likely change in the future, thus recommendations being provided may not be applicable in the long term.

### The Italian Scenario

The first cases of COVID-19 were reported in Italy on February 21st, 2020; one of them was in Padova and its province experienced a progressive lock-down that culminated with the nationwide lock-down of the whole Italian country on March 11th, 2020. Up to April 12th, the total number of positive cases in Veneto region was 14.170 with 869 confirmed deaths (3). Padova is the site of an academic Regional and National Referral Center, where dedicated COVID-19 wards were created, together with new ICU beds. Similarly to what has been described in other Countries (4), regional regulations disposed that hospitals should suspend non-oncological elective surgeries requiring ICU care, non-urgent outpatient procedures and visits, and limit other non-urgent surgery (5).

## Experience in Padova

Similarly to what was described by Naspro et al. (6), our Urology Department, experienced a significative change in daily clinical and surgical practice. In accordance with these regulations and the requests to shift some personnel to new dedicated COVID-19 wards, we tailored a specific organization of our department with three main objectives: (1) Ensure patients' and staff's safety, avoiding the risk of increasing contagion; (2) Provide the highest degree of medical service to the patients in terms of quality and quantity; (3) Work in accordance of regulations and scientific evidence.

Likewise to what described by Ngoi et al. regarding oncological care in Singapore (7) and, weeks ago, the specific recommendations to urological care (8), we adopted a segregated model to maintain urological care in a non-COVID-free hospital. Herein, a brief description of our organization is provided:

### Teamwork

In order to maintain a high level of care, it should be mandatory to minimize loss of workforce due to a potential contagion, thus the daily organization of staff members was intended to keep different units segregated, namely those working in the operating room had minimal or no contact with those having relationships with the outpatients or the inpatients. Physicians and staff leave for non-urgent need was suspended and residents attended to their activities on scheduled separated shifts, according to their level of autonomy in order to maintain their exposure to contagion at minimum. Operating room coaching activities were suspended, face-to-face meeting were canceled and a triage sector was created in each section of the Department where family and personal history were collected together with symptoms and body temperature measurements according to the triage criteria presented by Huang et al. (9). All personnel working in our Department (Consultants, Residents, Nurses, Health support personal, and Secretaries) were routinely tested for SARS-CoV-2 infection with nasopharyngeal swab at 3-week intervals.

### Scheduled Surgery

In order to allow sustainability of a slower system and to reallocate resources (PPE devices, respirators, ICU beds, physicians, and nurses) to the emergent COVID cases, a marked reduction of scheduled surgical activities was planned. The rescheduling of surgical intervention was evaluated on a case-by-case setting, where oncological and patient-related risk factors where balanced within a board of experienced physicians and anesthesiologists. All patients allowed in the Department were screened with an interview on symptoms and possible recent contacts with confirmed or suspected COVID cases. In case of positive screening, patients were referred to the Infectious Diseases Department for further evaluation. Additionally, patients undergoing general anesthesia were screened with nasopharyngeal swab PCR test the day before surgery. Operating room staff was provided with adequate PPE equipment and management of operating room procedures was planned together with the anesthesiology team (10).

### In-Hospital Stay and Management of Suspected Cases

During their in-hospital stay, patients had to wear surgical masks and visiting people were reduced to only one single relative for those patients requiring continue assistance. Body temperature, vital signs and in particular oxygen saturation were routinely measured at least four times a day; every alteration concerning for COVID infection activated a protocol consisting in isolation of the patients, repetition of the COVID-19 test, chest X-ray, and urgent pneumological evaluation. Clinically suspected cases with negative test remained isolated until further diagnostic tests such as search of SARS-CoV-2 immunoglobulins in the serum or High-Resolution Chest CT scan were proven negative. All staff including ward nurses and secretaries were provided PPE equipment.

### Outpatient Procedures and Visits

According to regional regulations, non-urgent scheduled outpatient procedures and visit were canceled. Patients with scheduled appointments were contacted by nurses to triage their situation and needs and were given the option to suspend their visit in case of non-urgent needs or directly contact a physician through e-mail and phone call in uncertain cases. Those requiring evaluation in a short time (<10 days) had the opportunity of being evaluated by a dedicated urologist every day with no need of scheduling an appointment, similarly to those requiring urgent evaluation that could refer to our 24 h service through the Emergency Department. Currently, a fully functional remote web consultation, “virtual clinic,” similarly to what is proposed by Connor et al. (11), is not yet available in our Department. In fact, this option could be mostly effective when ensuring adequate patient privacy and objective documentation that still need to be implemented in the different software used by the Institutions. We believe that this can represent a valid option in some selected cases and will probably be implemented in our practice in the near future.

### Research and Education

The clinical activity of the 27 residents on duty in our Department was modified according to the criteria described before and in-person teaching and training activities were substituted with online activities. Multidisciplinary meetings and tumor boards were shifted on online platforms.

### Results Achieved

We prospectively collected data on surgical procedures, subclassified by diagnosis, and type of intervention. Every patients was followed for 3 weeks after discharge to record surgical complications according to Clavien-Dindo Classification (12) and COVID-19 infection. With the same criteria, in a retrospective way, were reviewed data on surgical activity in March 2019.

During March 2020, the COVID-19 pandemic showed its exponential growing curve in Italy and in particular in Padua with 2227 cases until March 31st, in such way stressing this model.

In Table 1 are provided the results of our activity in aforementioned periods. Despite a 19.5% reduction of the monthly surgical activity (214 total cases in 2020 vs. 266 in 2019), we were able to maintain similar case mix compared to last year in particular continuing to provide care to oncological patients and also to high-priority non-oncological patients. Major surgical complications (Clavien-Dindo ≥ 3) were similar in the 2 years (13 in 2020 vs. 16 in 2019). No cases of COVID-19 were identified among patients nor staff, in this time period. Similarly, no case of infection was reported by our health authorities in patients treated or evaluated at our Department within the 14-days of potential incubation.

**Table 1:** Characteristics of surgical procedures performed in the months of March 2019 and 2020.

		**Year**		***p*-value^a^**
		**2020 (*n* = 214)**	**2019 (*n* = 266)**	
Age, median (IQR)		65 (52–72)	67 (49–75)	0.867
Sex, *n* (%)	Male	168 (78.5)	212 (79.7)	0.749
	Female	46 (21.5)	54 (20.3)	
Oncologic, *n* (%)	Yes	104 (48.6)	137 (51.5)	0.527
	No	110 (51.4)	120 (48.5)	
Type of surgery, *n* (%)	Major^b^	78 (36.4)	95 (35.7)	0.868
	Endoscopy	136 (73.6)	161 (64.3)	
Type of major surgery, *n* (%) Total = 173	Robotics/VLS	29 (37.2)	27 (28.4)	0.221
	Open	49 (62.7)	68 (71.6)	

a Mann-Whitney U-Test for continuous variable, Pearson Chi-Square Test for categorical variables.

b *Major surgery is intended as: radical cystectomy, radical prostatectomy, adenectomy, nephrectomy, or nephroureterectomy, transperitoneal pelvic organ prolapse correction. Minor (day-hospital or ambulatory) surgeries were excluded*.

**Figure 1:**
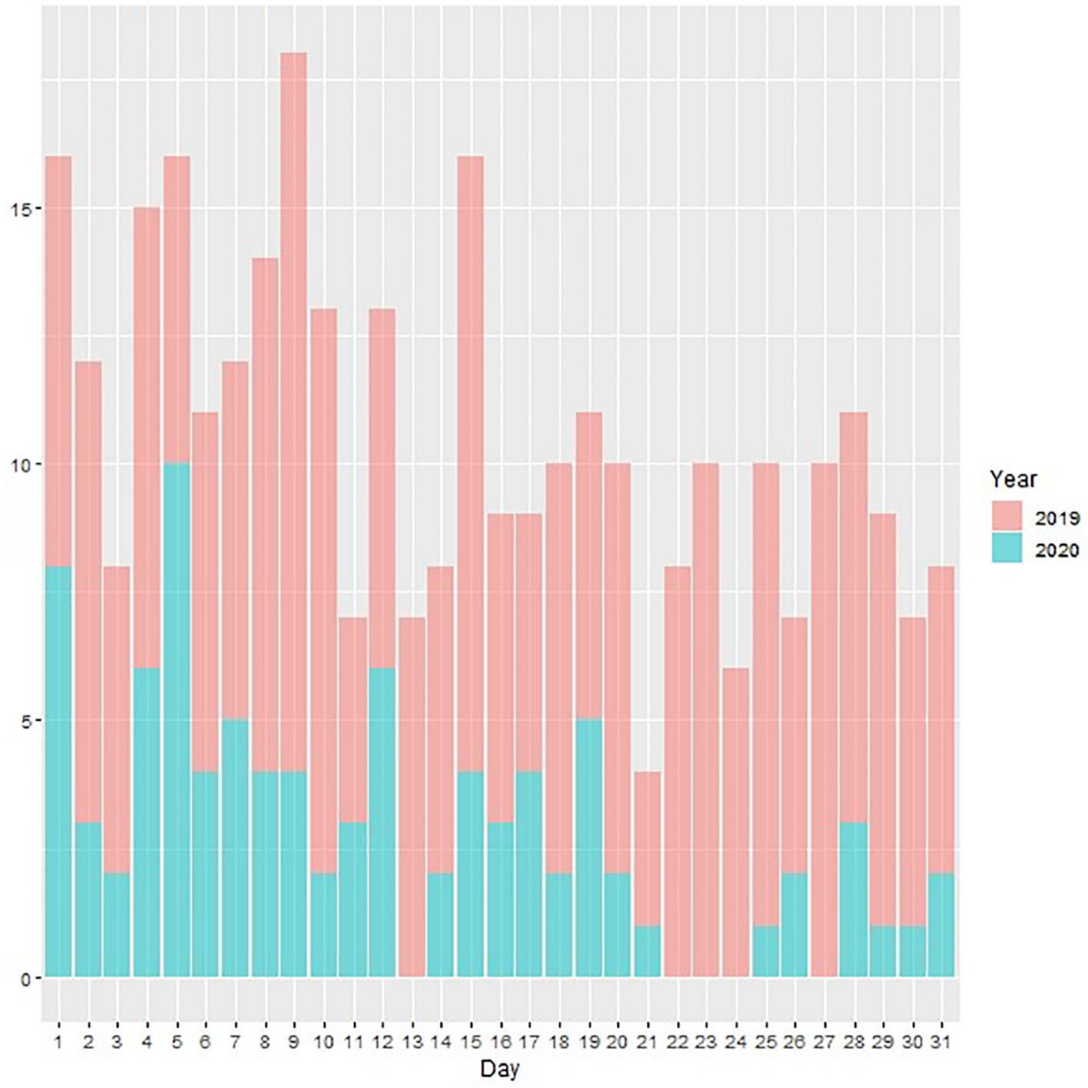
Plot of daily urgent consultations (Y axis) during the months of March 2019 and 2020 (X axis).

Urgent consultation activity and urgent admissions were maintained during this period of time. Despite an ~3-fold decrease in the median number of daily consultations requested by the Emergency Department (2.5 in 2020 vs. 7 in 2019, *p* < 0.001 in Mann-Whitney *U*-Test), there was a similar number of urgent admissions compared to last year (13 in 2020 vs. 15 in 2019) (Figure 1).

Moreover, the reorganization of the urological activities allowed a reduction of the beds commonly utilized in the ward, by ~50%. Likewise, the number of nurses on duty in the Operatory Room was reduced by 10% and by 20% the number of those on duty in the ward. The nurses removed from our service, were temporarily employed in the newly accomplished COVID-19 wards of the Hospital.

## Discussion

Although two coronaviruses had already caused severe respiratory syndromes previously in this century the severe acute respiratory syndrome coronavirus (SARS-CoV) and the Middle-East respiratory syndrome coronavirus (MERS-CoV), the current SARS-CoV-2 reached a pandemic spread and severely affected healthcare both at national and international level (1).

Consequently, a number of experts (8, 13–16) suggested the opportunity of a marked reduction of urological surgical activity, eventually leaving space only for urgent or oncological non-deferrable surgery during this COVID-19 emergency and recommending deferral of all the other activities.

On the other hand, Naspro et al. (6) recently highlighted the concern that any delay in surgery could affect the chances of patients of being effectively treated, including also a proportion of oncological patients. Similarly, the reduction in specialist consultations and diagnostic tests could cause an additional chain of delays in the diagnostic pathway of cancers (e.g., hematuria). Moreover, recent epidemiological models suggest that, after an initial pandemic explosion of COVID-19 contagion, there will be a long period of cohabitation with the virus in absence of a vaccine or specific therapy (2).

Given these considerations, a complete stop of urologic activities appears to be quite unrealistic, leading us to design a possible selective approach to urologic activities since the beginning of COVID-19 emergency. That could be effective even for the future cohabitation period, maintaining the highest level of care for the patients and a safe environment for healthcare professionals. Aware of these evidences and of the impossibility to work in a perfectly COVID-free environment, every effort must tend to maintain clinical activities minimizing the risk of infection through adequate patients' stratification and personnel protection as well as rationalizing elective surgeries with distinct pathways and surgical schedule re-arrangements and volume reduction.

### Specific Considerations About Surgery

Surgery in general is associated with increased levels of pro-inflammatory cytokines and a redistribution of lymphocytes, responsible for lymphopenia (17, 18). In addition, COVID-19 causes and abnormal pro-inflammatory cytokine response and lymphopenia (19). In this context, the risk of infection and complications must be adequately balanced with the risk of delaying an intervention until the end of the pandemic, which is actually still unknown. During March 2020, we performed number of so called minimally invasive (robotic or laparoscopic) interventions comparable to that of the same period of last year. Similarly, open surgical activity was maintained with only a mild reduction. There was no increase in significant surgical complications during the follow-up period.

Laparoscopic surgery, particularly, is a matter of debate as some Authors highlighted the potential higher risk of infection due to pneumoperitoneum-associated aerosolization of particles (20, 21) as well as the presence of the virus in blood and stool (22). To date however there is no evidence of contagion through these routes, nor laparoscopy gas has been associated with increased risk of infection with other viruses such as influenza and HIV (23). Emphasis (24, 25) is based on previous studies on papillomavirus but, to the best of our knowledge, there is no evidence of any association between SARS-CoV-2 and papillomavirus. Additionally, modern technologies of surgical gas recirculation such as CONMED Air-Seal®, that are extensively used in our Institution, guarantee a filtered suction of gas using ultralow particulate air filters (ULPA) rated to screen particles of 0.1 micron in diameter, which differ significantly from old laparoscopic instrumentation and protect operatory room staff from potential contamination (26).

Giving up laparoscopy means renouncing also the benefits connected to it, such as reduction of complications and length of stay (27) which seems counterproductive especially in this particular time and in those fragile patients that might benefit the most from minimally invasive surgery (28). In our experience we did not stop or reduce laparoscopic and robotic surgery without experiencing any infectious complication nor in patients and staff.

### Management Implications

Good managers are those with a clear vision of the future, even in this time when future seems still far. In addition to the described unknown effects of the virus, eventually we will face also the effects of our decisions. Provided that at the end of the COVID-19 pandemics our workforce will not be greater than before, we must be ready to face two new conditions. First, there will be a probably overwhelming overload of requests for those non-urgent conditions that have been delayed and this appear to be unmanageable with the previous allocation of resources, and that might require a completely new organization of local systems. Secondly, we will probably see an increase in proportion of advanced oncological cases; it is likely that, despite evidence supporting the safety in delay some oncological surgery as suggested by experts (15), the chain of delays concatenating the phases of diagnosis, staging, and treatment caused both by healthcare and patient-related factors will translate in delays extending further than the expected 3 months in the concrete real-life scenarios. From a general management perspective, the adaptation of the urological activities allowed to move a significant number of nurses to the temporary special COVID-19 wards in such a way supporting the Hospital general efforts in facing more effectively the pandemic.

### Final Considerations

The results herein presented are not exempt from limitations. The described data represent a picture of our experience and thus it has no power to prove any cause-effect relationship; additionally, it represents an experience from a single center in a limited period of time and thus may not be universally applicable. However, despite the raw numbers and results, to date there is a lack of evidence on what might be the best strategy to maintain urological care in this potentially long-lasting scenario.

In addition to that, it's still unclear the possible direct SARS-CoV-2 impact on urogenital system (29, 30). Hereafter it might be plausible that even mild effects of the virus on the urogenital system will cause a non-negligible burden of impact on our urological daily practice.

The experience from a Referral Center in Italy, hardly hit by COVID-19 pandemic, that maintained its activity splitting non-COVID from COVID Patients, might be useful and provides interesting topics for further discussion.

## Conclusions

Reliable evidence on these topics of discussion is still far from being available and, to the best of our knowledge, current recommendations consist in Level 5 evidence (i.e., expert opinion). Our paper represents one of the first reported real-life experiences in the urological setting from a high-volume Referral Center facing the COVID-19 pandemics and will provide elements for planning urological care during of potentially long-lasting a period of cohabitation with COVID infection.

## Data Availability Statement

The raw data supporting the conclusions of this article will be made available by the authors, without undue reservation.

## Author Contributions

TP-G, DB, and FZ: conception and design. GM, FC, and AM: acquisition of data. GM, FC, and MI: analysis and interpretation of data. GM: statistical analysis. TP-G, GM, and AM: manuscript writing/editing. FZ, TP-G, MI, and DB: supervision. All authors contributed to the article and approved the submitted version.

## Conflict of Interest

The authors declare that the research was conducted in the absence of any commercial or financial relationships that could be construed as a potential conflict of interest.

## References

[1] WHO Novel Coronavirus. (2020). Available online at: https://www.who.int/emergencies/diseases/novel-coronavirus-2019 (accessed April 1, 2020)

[2] Kissler SM Tedijanto C Goldstein E Grad YH Lipsitch M. Projecting the transmission dynamics of SARS-CoV-2 through the postpandemic period. Science. (2020) 368:860–8. 10.1126/science.abb5793 32291278 PMC7164482

[3] Ministero Della Salute Nuovo Coronavirus. (2020). Available online at: http://www.salute.gov.it/nuovocoronavirus (accessed April 12, 2020)

[4] Iacobucci G. Covid-19: all non-urgent elective surgery is suspended for at least three months in England. BMJ. (2020) 368:m1106. 10.1136/bmj.m1106 32188602

[5] Regione Veneto Coronavirus. Il Veneto Riorganizza Attività Sanitarie per Preservare Posti di Letto di Terapia Intensiva. (2020). Available online at: https://www.regione.veneto.it/article-detail?articleId=4377038 (accessed April 1, 2020)

[6] Naspro R Da Pozzo LF. Urology in the time of corona. Nat Rev Urol. (2020) 17:251–3. 10.1038/s41585-020-0312-1 32203310 PMC7095235

[7] Ngoi N Lim J Ow S Jen WY Lee M Teo W. A segregated-team model to maintain cancer care during the COVID-19 outbreak at an academic center in Singapore. Ann Oncol. (2020) 31:840–3. 10.1016/j.annonc.2020.03.306 32243893 PMC7174823

[8] Ribal MJ Cornford P Briganti A Knoll T Gravas S Babjuk M. European Association of Urology Guidelines Office Rapid Reaction Group: An organisation-wide collaborative effort to adapt the European Association of Urology Guidelines Recommendations to the Coronavirus Disease 2019 era. Eur Urol. (2020) 78(1):21-28. 10.1016/j.eururo.2020.04.056 32376137 PMC7183974

[9] Huang C Wang Y Li X Ren L Zhao J Hu Y. Clinical features of patients infected with 2019 novel coronavirus in Wuhan, China. Lancet. (2020) 395:497–506. 10.1016/S0140-6736(20)30183-5 31986264 PMC7159299

[10] Grieco DL Anzellotti GM Russo A Bongiovanni F Costantini B D'Indinosante M. Airway closure during surgical pneumoperitoneum in obese patients. Anesthesiol J Am Soc Anesthesiol. (2019) 131:58–73. 10.1097/ALN.0000000000002662 30882475

[11] Connor MJ Winkler M Miah S. COVID-19 Pandemic – Is Virtual Urology Clinic the answer to keeping the cancer pathway moving? BJU Int. (2020) 125:E3–E4. 10.1111/bju.15061 32232915

[12] Dindo D Demartines N Clavien P-A. Classification of surgical complications: a new proposal with evaluation in a cohort of 6336 patients and results of a survey. Ann Surg. (2004) 240:205–13. 10.1097/01.sla.0000133083.54934.ae 15273542 PMC1360123

[13] Simonato A Giannarini G Abrate A Bartoletti R Crestani A De Nunzio C. Pathways for urology patients during the COVID-19 pandemic. Minerva Urol Nefrol. (2020) 72:376–83. 10.23736/S0393-2249.20.03861-8 32225135

[14] Ficarra V Novara G Abrate A Bartoletti R Crestani A De Nunzio C. Urology practice during COVID-19 pandemic. Minerva Urol Nefrol. (2020) 72:369–75. 10.23736/S0393-2249.20.03846-1 32202401

[15] Stensland KD Morgan TM Moinzadeh A Lee CT Briganti A Catto JWF. Considerations in the Triage of Urologic Surgeries During the COVID-19 Pandemic. Eur Urol. (2020) 77: 663–6. 10.1016/j.eururo.2020.03.027 32279903 PMC7146681

[16] Goldman HB Haber GP. Recommendations for tiered stratification of urologic surgery urgency in the COVID-19 era. J Urol. (2020) 204:11–3. 10.1097/JU.0000000000001067 32249715 PMC7273865

[17] Lin E Calvano SE Lowry SF. Inflammatory cytokines and cell response in surgery. Surgery. (2000) 127:117–26. 10.1067/msy.2000.101584 10686974

[18] Corral-Velez V Lopez-Delgado JC Betancur-Zambrano NL Lopez-Sune N Rojas-Lora M Torrado H. The inflammatory response in cardiac surgery: an overview of the pathophysiology and clinical implications. Inflamm Allergy Drug Targets. (2015) 13:367–70. 10.2174/1871528114666150529120801 26021321

[19] Besnier E Tuech J-J Schwarz L. We asked the experts: Covid-19 outbreak: is there still a place for scheduled surgery? Reflection from Pathophysiological Data. World J Surg. (2020) 44:1695–8. 10.1007/s00268-020-05501-6 32246185 PMC7124188

[20] Mottrie A ERUS (EAU Robotic Urology Section) Guidelines During COVID-19 Emergency. (2020). Available online at: https://uroweb.org/eau-robotic-urology-section-erus-guidelines-during-covid-19-emergency/

[21] Cohen SL Liu G Abrao M Smart N Heniford T. Perspectives on surgery in the time of COVID-19: safety first. J Minim Invasive Gynecol. (2020) 27:792–3. 10.1016/j.jmig.2020.04.003 32251839 PMC7129781

[22] Zhang W Du R-H Li B Zheng X-S Yang X-L Hu B. Molecular and serological investigation of 2019-nCoV infected patients: implication of multiple shedding routes. Emerg Microbes Infect. (2020) 9:386–9. 10.1080/22221751.2020.1729071 32065057 PMC7048229

[23] Morris SN Fader AN Milad MP Dionisi HJ. Understanding the scope of the problem: why laparoscopy is considered safe during the COVID-19 pandemic. J Minim Invasive Gynecol. (2020) 27:789–91. 10.1016/j.jmig.2020.04.002 32247882 PMC7129473

[24] Sawchuk WS Weber PJ Lowy DR Dzubow LM Infectious papillomavirus in the vapor of warts treated with carbon dioxide laser or electrocoagulation: detection and protection. J Am Acad Dermatol. (1989) 21:41–9. 10.1016/s0190-9622(89)70146-8 2545749

[25] Gloster HMJ Roenigk RK. Risk of acquiring human papillomavirus from the plume produced by the carbon dioxide laser in the treatment of warts. J Am Acad Dermatol. (1995) 32:436–41. 10.1016/0190-9622(95)90065-9 7868712

[26] CONMED AirSeal^®^ *iFS*. Available online at: https://www.conmed.com/it/medical-specialties/laparoscopic-robotic-and-open-surgery/general-and-bariatric-surgery/access/airseal-system/airseal-products/airseal-ifs-intelligent-flow-system (accessed April 16, 2020)

[27] Ploussard G. Robotic surgery in urology: facts and reality. What are the real advantages of robotic approaches for prostate cancer patients? Curr Opin Urol. (2018) 28:153–8. 10.1097/MOU.0000000000000470 29232271

[28] Gallotta V Conte C D'Indinosante M Federico A Biscione A Vizzielli G Robotic surgery in elderly and very elderly gynecologic cancer patients. J Minim Invasive Gynecol. (2018) 25:872–7. 10.1016/j.jmig.2018.01.007 29339300

[29] Puliatti S Eissa A Eissa R Amato M Mazzone E Dell'Oglio P. COVID-19 and urology: a comprehensive review of the literature. BJU Int. (2020) 125:E7–14. 10.1111/bju.15071 32249538

[30] Wu Z-S Zhang Z-Q Wu S. Focus on the crosstalk between COVID-19 and urogenital systems. J Urol. (2020) 204:7–8. 10.1097/JU.0000000000001068 32249664 PMC7273857

